# Supplementary material for: GP73 is a TBC-domain Rab GTPase-activating protein contributing to the pathogenesis of non-alcoholic fatty liver disease without obesity
Source: Nat Commun. 2021 Dec 1;12:7004. doi: 10.1038/s41467-021-27309-1 (PMC8636488; doi:10.1038/s41467-021-27309-1)
Supplement: Supplementary file 1 — Supplementary information. [file 41467_2021_27309_MOESM1_ESM.pdf]

Supplementary Information

GP73 is a TBC-domain Rab GTPase-activating protein contributing to the pathogenesis of non-alcoholic fatty liver disease without obesity

Yumeng Peng[1,2,8], Qiang Zeng[3,8], Luming Wan[1,8], Enhao Ma[4,8], Huilong Li[1,2,8], Xiaopan Yang[1,8], Yanhong Zhang[1], Linfei Huang[1], Haotian Lin[1], Jiangyue Feng[1], Yixin Xu[1,2], Jingfei Li[1], Muyi Liu[1], Jing Liu[1], Changqin Lin[5], Zhiwei Sun[5], Gong Cheng[4], Xuemiao Zhang[1,6], Jialong Liu[1,6], Dongrui Li[1], Meng Wei[1,2], Yunhai Mo[1,2], Xuetao Mu[6], Xiaowei Deng[6], Dandan Zhang[6], Siqing Dong[6], Hanqing Huang[1], Yi Fang[7]*, Qi Gao[5]*, Xiaoli Yang[6]*, Feixiang Wu[2]*, Hui Zhong[1]*, and Congwen Wei[1]*

[1] Beijing Institute of Biotechnology, Academy of Military Medical Sciences (AMMS), Beijing, China.

[2] Department of Hepatobiliary Surgery, Affiliated Tumor Hospital of Guangxi Medical University, Nanning, China.

[3] Health management Institute, The Second Medical Center and National Clinical Research Center for Geriatric Diseases, Chinese PLA General Hospital, Beijing, China.

[4] Tsinghua-Peking Center for Life Sciences, School of Medicine, Tsinghua University, Beijing, China.

[5] Beijing Sungen Biomedical Technology Co., Ltd., Beijing, China.

[6] Department of Clinical Laboratory, the Third Medical Center, Chinese PLA General Hospital, Beijing, China.

[7] Department of Endocrinology, the Fifth Medical Centre, Chinese PLA General Hospital, Beijing, China.

[8] These authors contributed equally to this work: Yumeng Peng, Qiang Zeng, Luming Wan, Enhao Ma, Huilong Li, Xiaopan Yang.

*These authors jointly supervised the work: Yi Fang, Qi Gao, Xiaoli Yang, Feixiang Wu, Hui Zhong, Congwen Wei.

Supplementary Figures

Supplementary Fig.1

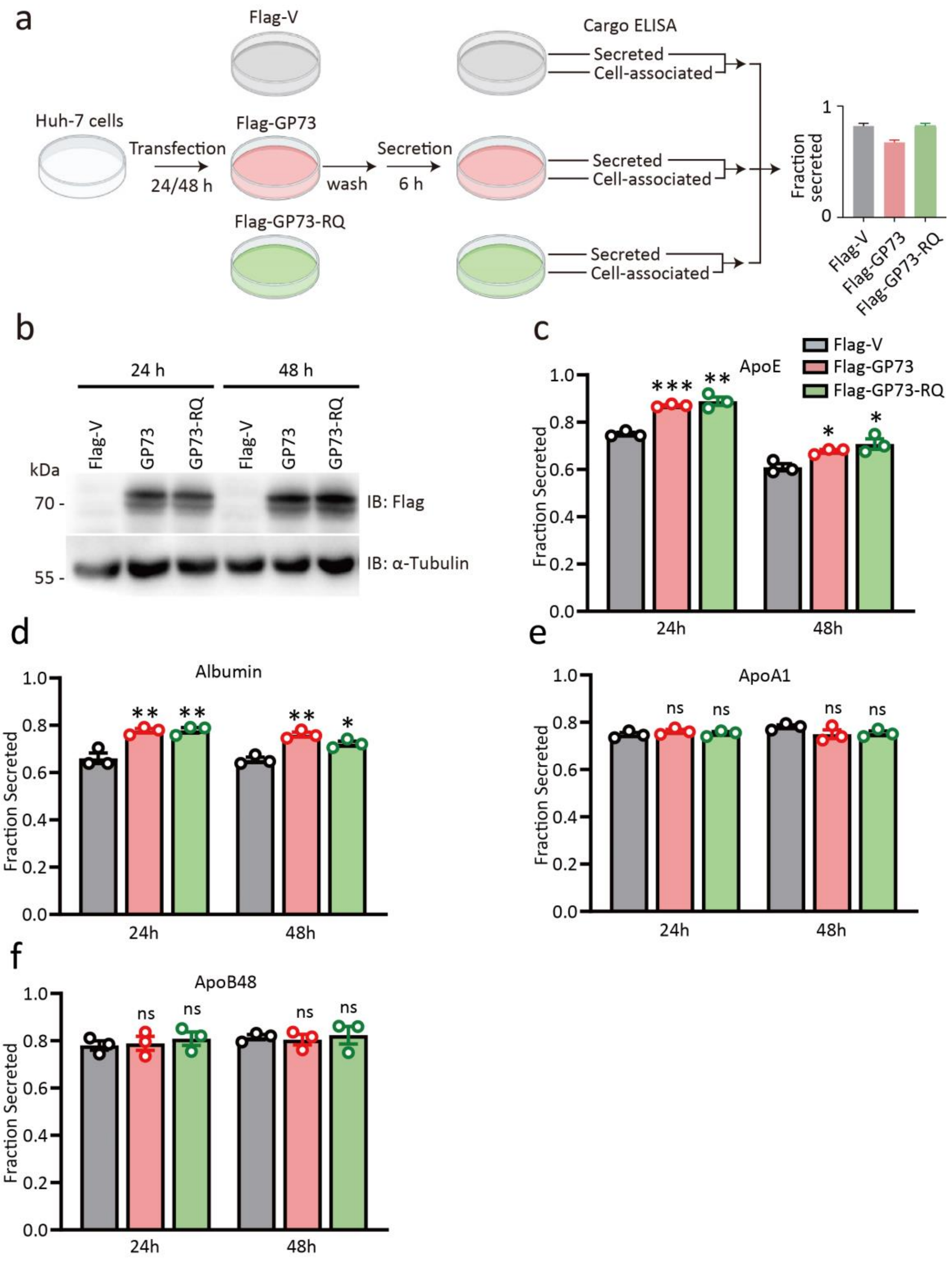

Supplementary Fig. 1 GP73 reduces ApoB secretion in a GAP activity-dependent manner (related to Fig. 1). a Screening outline. Huh-7 cells transfected with Flag-vector (Flag-V), Flag-GP73, or Flag-GP73-RQ mutant were washed 24 or 48 h

after transfection and allowed to secrete cargo in fresh medium for 6 h. The amounts of cargo that were secreted into the medium or that remained cell-associated were detected by ELISA in both medium and cell lysates. The secretion efficiency fraction was calculated as the ratio between the amount of cargo that was secreted and the total amount of cargo (secreted plus cell-associated cargo). **b** GP73 protein expression in Huh-7 cells transfected with Flag-vector (Flag-V), Flag-GP73, or Flag-GP73-RQ. α-Tubulin was used as the equal loading control. Data were repeated three times with similar results. **c-f** ApoE **(c)**, albumin **(d)**, ApoA1 **(e),** and ApoB48 **(f)** levels in both the medium and cell lysates from cells transfected with Flag-vector (Flag-V), Flag-GP73, or Flag-GP73-RQ mutant at the indicated time points after transfection. n = 3 independent biological experiments. Differences between two groups were evaluated using the unpaired Student's t-test. Data were presented as mean values ± SEM. ns, no statistical significance; *P < 0.05; **P < 0.01; ***P < 0.001.

Supplementary Fig. 2

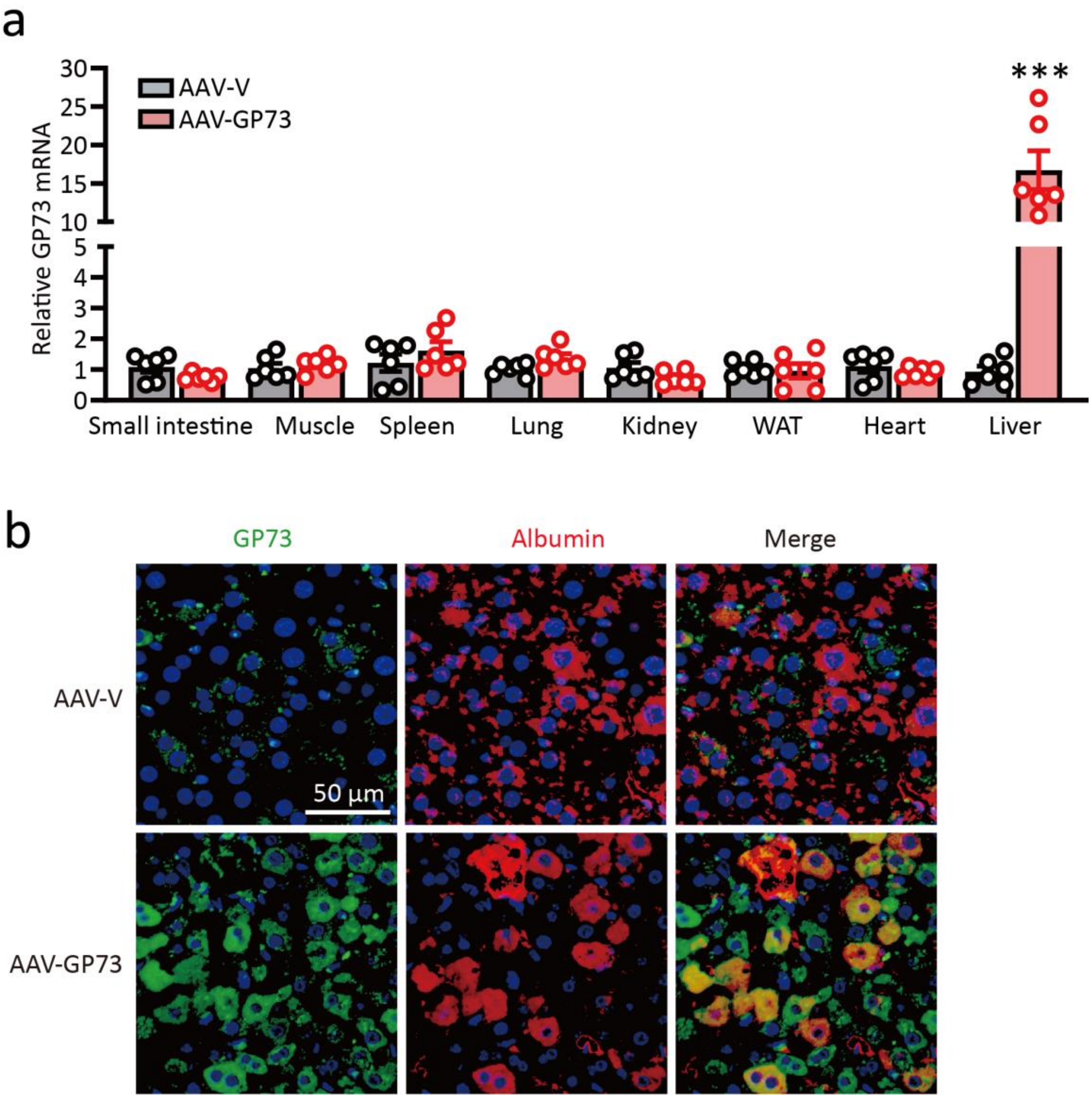

Supplementary Fig. 2 AAV-GP73 induced GP73 expression is restricted to liver tissues (related to Fig. 1). a GP73 mRNA expression in multiple tissues from AAV-V- or AAV-GP73-injected mice fed a regular diet for 6 months (n = 6 per group). Differences between two groups were evaluated using the unpaired Student's t-test. Data were presented as mean values ± SEM. ***P < 0.001.

b GP73 and albumin staining of livers from AAV-V- or AAV-GP73-injected mice fed a regular diet for 6 months (n = 6 per group).

Supplementary Fig. 3

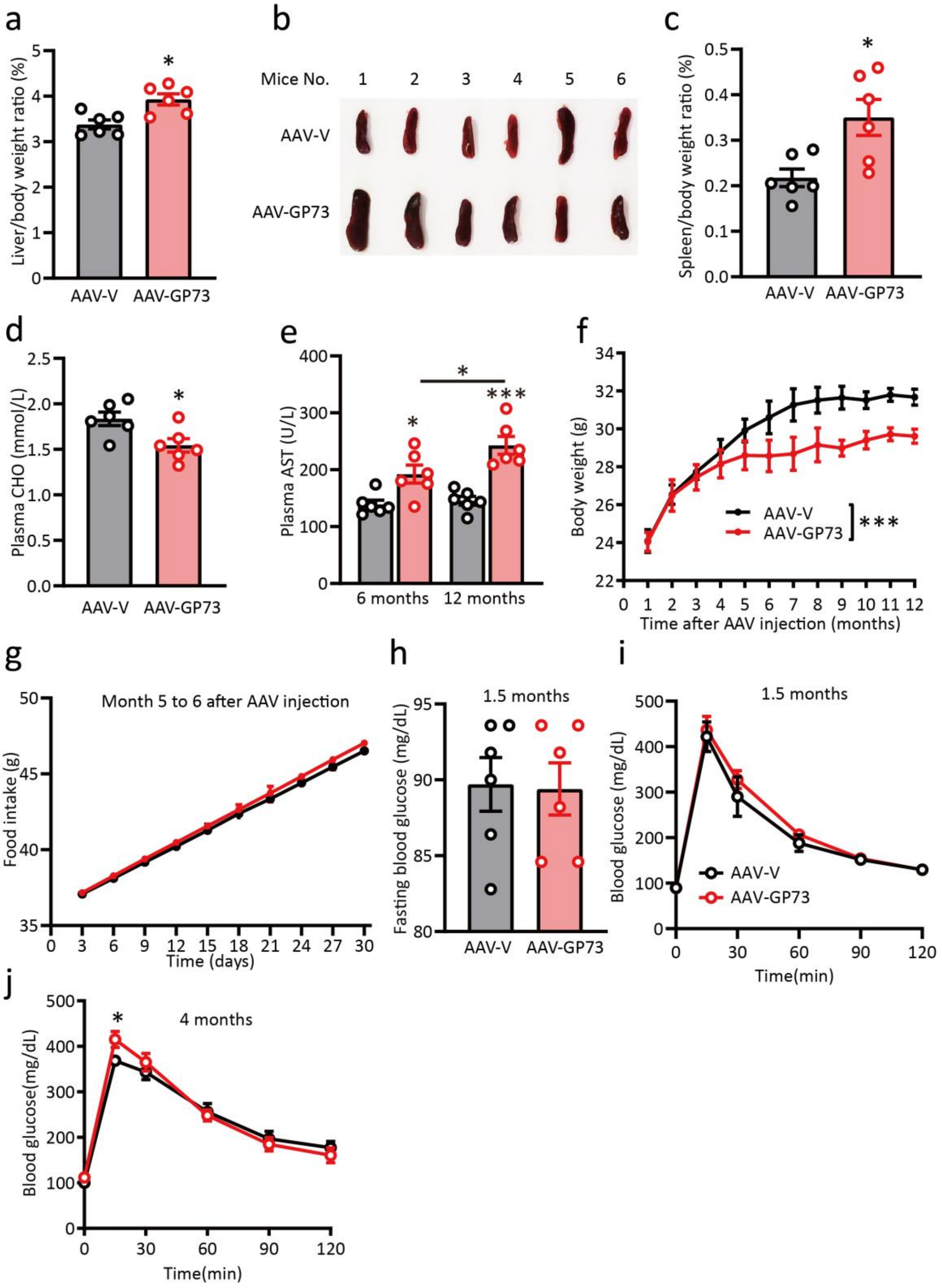

Supplementary Fig. 3 Chronic elevations in hepatocyte GP73 trigger non-obese NAFLD (related to Fig. 2). a-c Liver-to-body weight ratio **(a)**, spleens **(b)**, and spleen-to-body weight ratio (**c**; n = 6 per group) of the AAV-V- or AAV-GP73-injected

mice fed a regular diet for 6 months. Differences between two groups were evaluated using the unpaired Student's t-test. Data were presented as mean values ± SEM. *P < 0.05. **d,e** Plasma levels of CHO **(d)** and AST **(e)** in AAV-V- or AAV-GP73-injected mice fed a regular diet for 6 or 12 months (n = 6 per group). Differences between two groups were evaluated using the unpaired Student's t-test. Data were presented as mean values ± SEM. *P < 0.05; ***P < 0.001. **f,g** Body weight (f) and food intake (g) of AAV-V- or AAV-GP73-injected mice fed a regular diet at the indicated times (n=6). Data of food intake were presented as the amount of cumulative food eaten by 2 mice per cage. ***P = 0.0002 by two-way ANOVA. Data were presented as mean values ± SEM. **h** Glucose levels in blood samples from 6-h-fasted AAV-V- or AAV-GP73-injected mice at 1.5 months after injection (n = 6 per group). Differences between two groups were evaluated using the unpaired Student's t-test. Data were presented as mean values ± SEM. **i,j** Glucose tolerance test (GTT) for AAV-V- or AAV-GP73-injected mice at 1.5 months **(i)** and 4 months **(j)** after injection (n = 6 per group). Differences between two groups were evaluated using two-way ANOVA and Bonferroni's post hoc analysis. Data were presented as mean values ± SEM. *P < 0.05.

Supplementary Fig. 4

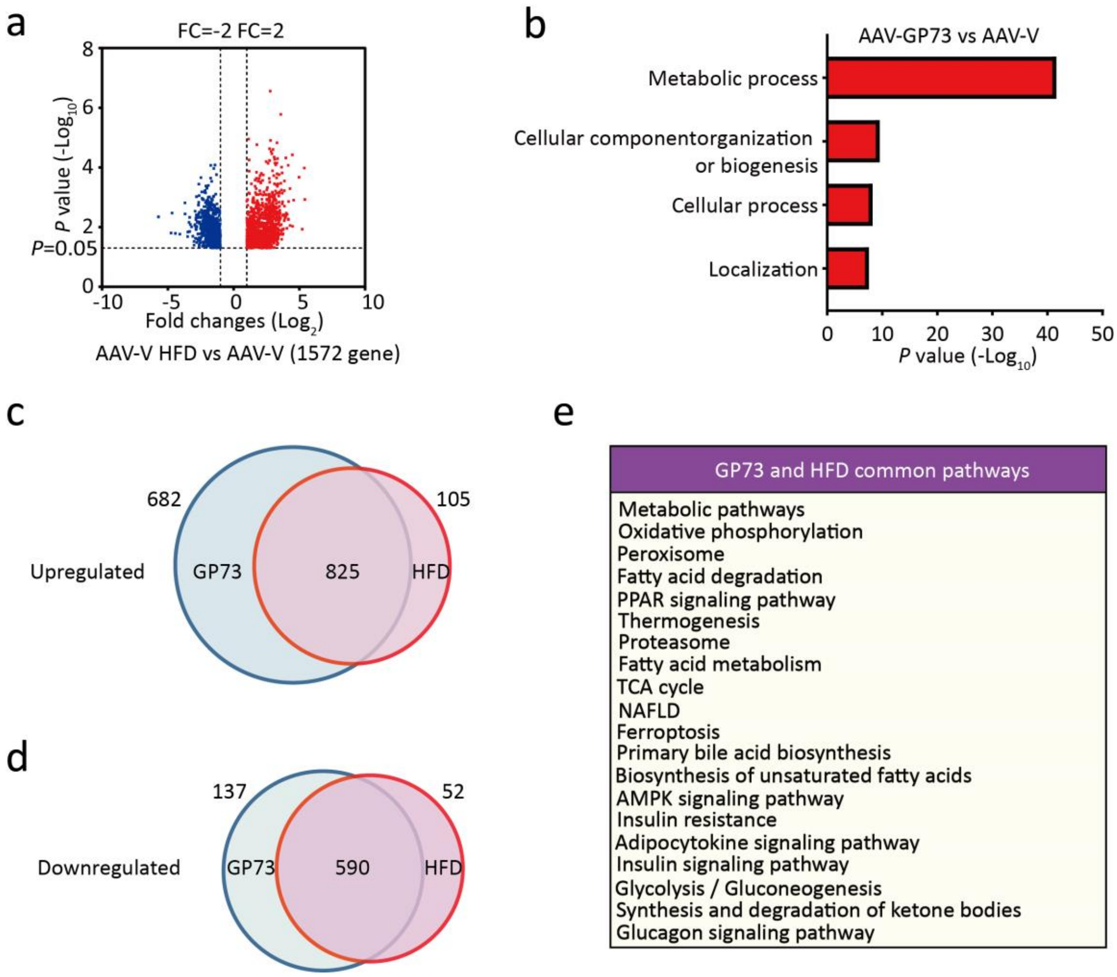

Supplementary Fig. 4 Gene expression signatures in non-obese NAFLD induced by GP73 (related to Fig. 4). a Volcano plot of the DEGs in the livers from AAV-V-injected mice fed a HFD for 12 months (n = 3 per group). Significantly downregulated genes are in blue, and significantly upregulated genes are in red. The data were analyzed with two-sided Student's t-test. The black vertical lines highlight fold changes (FCs) of -2 and 2, while the black horizontal line represents a *P* value of 0.05. **b** Pathways enriched for the DEGs in the livers from AAV-GP73-injected mice at 12 months after injection according to GO term analysis at GO level 2 (n = 3 per group). The bar plot shows significantly dysregulated pathways, and Fisher's exact test P values shown on the x-axis. **c-d** Map of the upregulated genes **(c)** and downregulated genes **(d)** in the

livers from AAV-GP73 mice versus HFD mice. **e** Pathways commonly enriched in both the AAV-GP73 and HFD groups according to KEGG pathway analyses.

Supplementary Fig. 5

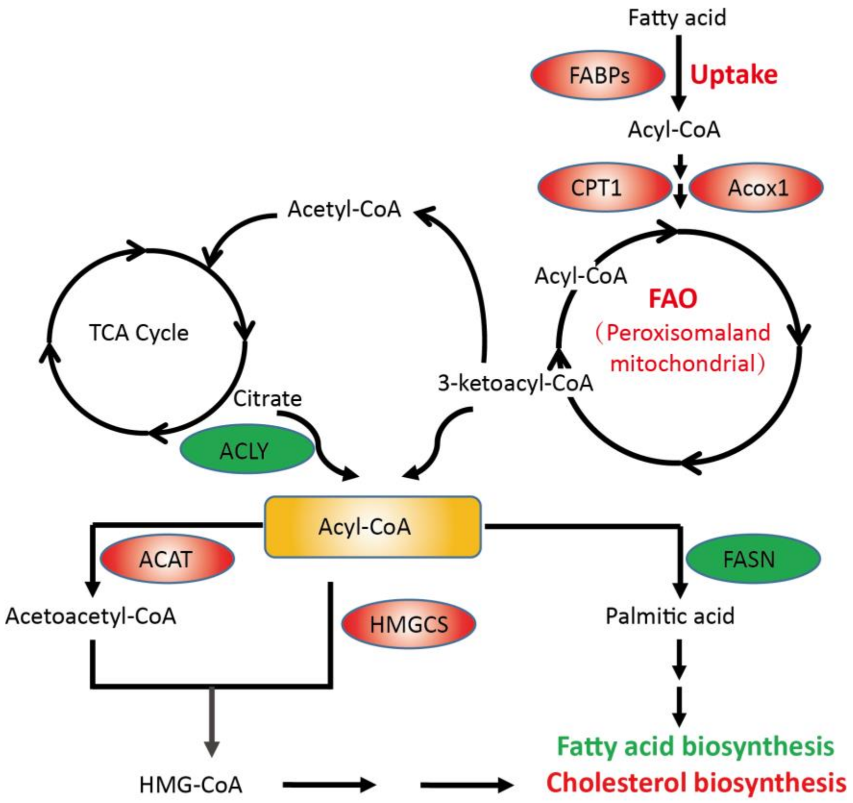

Supplementary Fig. 5 Gene expression signatures in non-obese NAFLD induced by GP73 (related to Fig.4). DEGs of the critical enzymes in the livers from AAV-GP73-injected mice versus AAV-V-injected mice fed a regular diet for 12 months. Upregulated genes and pathways are highlighted in red, and downregulated genes and pathways are highlighted in green.

Supplementary Fig. 6

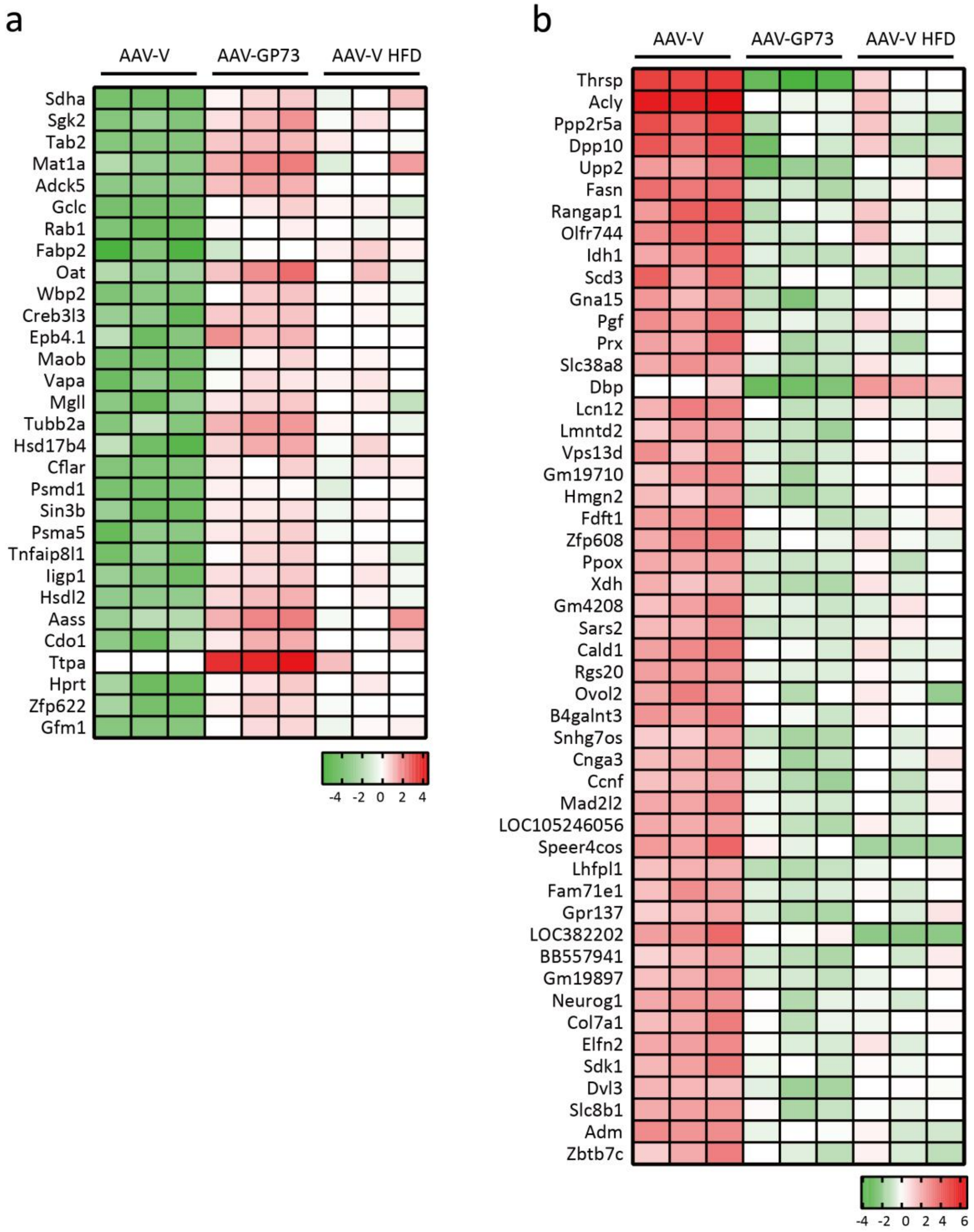

Supplementary Fig. 6 Gene expression signatures were similar in non-obese NAFLD induced by GP73 and obese NAFLD induced by HFD (related to Fig. 4). a,b Heatmap of the top 21-50 highly upregulated genes (a) and the top 50 highly downregulated genes (b) in the livers from the AAV-GP73 and HFD groups.

Supplementary Fig. 7

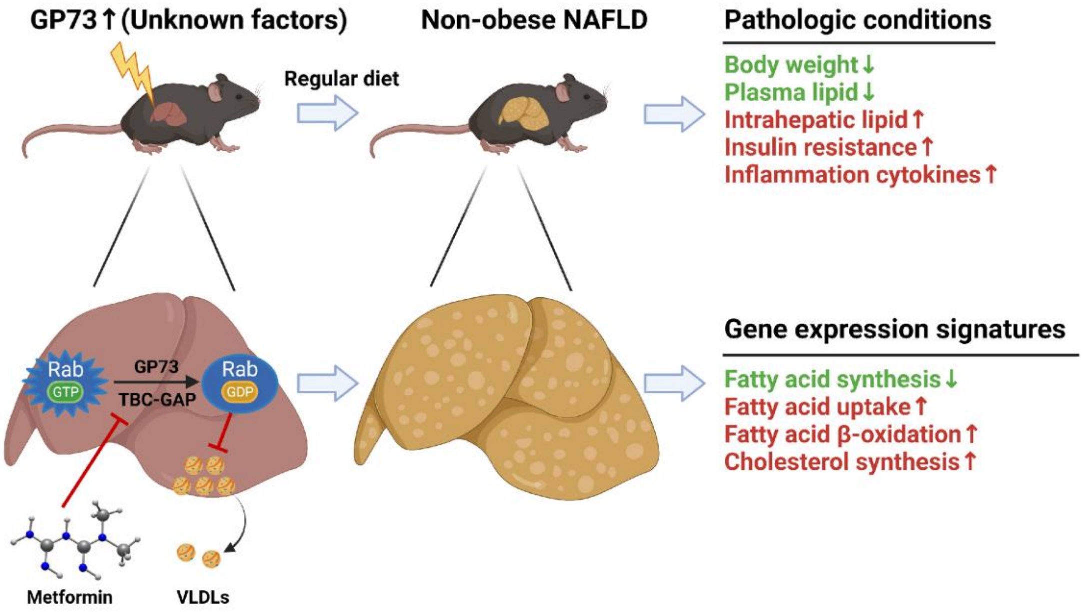

Supplementary Fig. 7 Hypothetical model of the involvement of GP73 in triggering non-obese NAFLD. In the context of the consumption of a regular diet, the prolonged increase in hepatocyte GP73 induced by unknown factors will lead to the onset of non-obese NAFLD, including reduced body weight, decreased serum lipid levels, massive intrahepatic lipid accumulation, elevated baseline levels of inflammatory cytokines, and gradual insulin resistance development. Transcriptional changes in GP73-high livers displayed enhanced FAO activity, reduced FA synthesis, upregulated FA uptake and increased cholesterol synthesis. The figure was created and exported with BioRender.com under a paid subscription.

Supplementary Table

Supplementary Table 1. Characteristics of the study participants based on obesity status.

Characteristics	Non-obese controls (n=14)	NAFLD without obesity (n=14)	*P* value
Age (years)	39.2±13.6	40.1±10.4	0.8456
Gender (male, %)	10 (71.4)	9 (64.3)	
BMI (kg/m^2)	21.5±3.5	23.5±1.4	0.0578
WC (cm)	79.5±6.5	81.3±8.4	0.5376
Glucose (mmol/L)	5.1±0.5	5.4±1.7	0.5320
SBP (mmHg)	116.4±9.0	121.5±54.2	0.7311
DBP (mmHg)	68.0±6.6	69.6±13.8	0.6987
WBC (x10^9/L)	5.3±1.0	6.8±3.3	0.1157
Hb (g/L)	127.9±15.8	144.5±64.0	0.3548
PLT (x10^9/L)	227.5±41.1	259.6±130.1	0.3868
TG (mmol/L)	0.7±0.2	1.9±1.6	0.0099
CHO (mmol/L)	4.9±0.7	4.8±0.9	0.7454
LDL-C (mmol/L)	2.6±0.6	2.6±0.7	$>$0.9999
HDL-C (mmol/L)	1.7±0.3	1.2±0.4	0.0009
ALT (U/L)	16.7±7.3	37.6±28.8	0.0141
AST (U/L)	21.5±17.8	24.2±9.8	0.6232
UA (μmol/L)	305.1±74.9	426.9±85.3	0.0004
Adiponectin (μg/mL)	8.0±2.4	5.0±1.3	0.0004
Leptin (ng/mL)	7.7±3.1	8.5±2.9	0.4870
A/L ($\times10^3$)	1.3±1.0	0.7±0.3	0.0302
MRI-HFF (%)	3.0±2.4	32.1±14.8	$<$0.0001

Abbreviations: NAFLD, nonalcoholic fatty liver disease; BMI, body mass index; WC, waist circumference; SBP, systolic blood pressure; DBP, diastolic blood pressure; WBC, white blood cell; Hb, hemoglobin; PLT, platelet; TG, triglyceride; CHO, cholesterol; HDL, high-density lipoprotein; LDL, low-density lipoprotein; ALT, alanine aminotransferase; AST, aspartate aminotransferase; UA, uric acid; A/L, adiponectin to leptin ratio; MRI-HFF, magnetic resonance imaging hepatic fat fraction.
The data are expressed as the means ± SDs or numbers (percentages).
The statistical analyses (*P* value) were performed by comparing non-obese controls vs. NAFLD without obesity by two-sided unpaired Student's t-tests.

Supplementary Methods

Magnetic Resonance Imaging (MRI)

The MRI examinations were performed with a 3.0T MRI imaging system (Trio Tim Siemens, Germany) with an 8-channel body coil. Dual in-phase, opposed-phase T1-based gradient echo image acquisition was performed in the axial plane during an end-expiratory breath-hold with an approximate acquisition time of 16 s. The two-point Dixon method based on phase-shift imaging was used in which hepatic fat fraction (HFF) was calculated by computing the relative signal intensity (SI) decrease in the liver on opposed-phase images compared with in-phase images after taking a mean of twelve >1 cm^2 regions of interest (ROIs) placed on multiple slices, taking care to avoid areas with vessels, motion artifacts, and partial volume effects. ROIs were placed at anatomically matched locations on paired images by using a coregistration tool available on the workstation to ensure assessment of similar liver parenchyma on in- and out-phase images. Because the tissue of interest is measured at a colocalized location at each TE, depth-dependent SI changes in the image do not confound the results[1].

The dual-echoT1-weighted sequence parameters were as follows: repetition time of 290 ms; echo time of 1.2 ms for OP images and 2.3 ms for IP images; flip angle, 70°; section thickness, 6 mm; matrix size, 288 × 188; FOV, 34 cm × 45 cm. HFF was calculated as the percentage of relative SI loss of the liver on opposed-phase images compared to in-phase images, with the following formula: $HFF = [(SIin - SIout)/2 \times SIin] \times 100$, where SIin and SIout are SI of IP and OP images, respectively. MR imaging

results were interpreted by an experienced radiologist who was blinded to the clinical, laboratory, and histological findings[2]. The diagnosis of NAFLD was based on magnetic resonance imaging (MRI) with HFF ≥ 5.5%. The control group with < 5.5% HFF, and the fatty liver group comprised patients with ≥ 5.5% HFF[3].

GAP assay

A GAP assay using an EnzChek Phosphate Assay Kit (Invitrogen, E12020) and kinetics determinations were performed in strict accordance with a previously described procedure[4]. Briefly, Rabs were loaded with GTP (Thermo Fisher Scientific, R0461) by incubating GP73 with a 50-fold molar of GTP at 25 °C for 1 h in 20 mM HEPES pH 7.5, 150 mM NaCl, 5 mM EDTA, and 1 mM dithiothreitol. Free GTP was removed with a desalting column (Thermo Fisher Scientific, 89891) pre-equilibrated with 20 mM HEPES pH 7.5 and 150 mM NaCl. The single-turnover kinetics of intrinsic and GAP-accelerated GTP hydrolysis were measured by a continuous enzyme assay for the release of inorganic phosphate with the use of reagents from the EnzChek Phosphate Assay Kit (Invitrogen, E12020). GTP-loaded Rabs were mixed with solutions containing the assay reagents and GAPs. The final solutions contained 20 mM HEPES pH 7.5, 150 mM NaCl, 0.15 mM 2-amino-6-mercapto-7-methylpurine ribonucleoside, 0.75 U/ml purine nucleoside phosphorylase, 10 mM $MgCl_2$, 20 nM GP73 protein and various concentrations of GTP-loaded Rabs. The absorbance at 360 nm was monitored with a microplate spectrometer (Tecan, M1000). The data were analyzed by fitting them simultaneously to the pseudo-first-order Michaelis-Menten model function:

$$A(t) = (A_\infty - A_0)\left(1 - e^{-\mathrm{kt}}\right) + A_0$$

$$k_{\mathrm{obs}} = k_{\mathrm{intr}} + \frac{k_{\mathrm{cat}}}{K_{\mathrm{M}}}[GAP]$$

To calculate the catalytic efficiency (k_{cat}/K_m), the observed kinetics (k_{obs}) and the intrinsic rate constant (k_{intr}) were measured by fitting the data into a linear regression model according to the transformation form of the pseudo-first-order Michaelis-Menten model function. The calculation is shown below:

$$A(t) = (A_\infty - A_0)\left(1 - e^{-\mathrm{kt}}\right) + A_0$$

$$A(t) = (A_\infty - A_0) - (A_\infty - A_0)e^{-\mathrm{kt}} + A_0$$

$$A(t) = A_\infty - (A_\infty - A_0)e^{-\mathrm{kt}}$$

$$A(t) - A_\infty = -(A_\infty - A_0)e^{-\mathrm{kt}}$$

$$A_\infty - A(t) = (A_\infty - A_0)e^{-\mathrm{kt}}$$

$$\ln[A_\infty - A(t)] = \ln[(A_\infty - A_0)e^{-\mathrm{kt}}]$$

$$\ln[A_\infty - A(t)] = \ln(A_\infty - A_0) + \ln(e^{-\mathrm{kt}})$$

$$\ln[A_\infty - A(t)] = \ln(A_\infty - A_0) - kt$$

From this equation, $\ln[A_\infty - A(t)]$ was regarded as the response variable and was regressed on the explanatory variable time t. The resulting regression coefficients were the desired rate constants with negative signs in the front. The observed kinetics (k_{obs}) and the intrinsic rate constant (k_{intr}) were then acquired by removing the minus signs, and the values of k_{obs} and k_{intr} were plugged back into the equation below to obtain the catalytic efficiency (k_{cat}/K_m) with the concentration of GTPase-activating protein (GAP) set to 20 nM:

$$k_{\mathrm{obs}} = k_{\mathrm{intr}} + \frac{k_{\mathrm{cat}}}{K_{\mathrm{M}}}[GAP]$$

The catalytic efficiency (k_{cat}/K_m) and intrinsic rate constant for GTP hydrolysis (k_{intr}) were treated as global parameters.

Microscale thermophoresis (MST) assay

An MST assay was conducted as previously described[5]. GP73-His-tagged proteins were labeled with NT-647 dye for 30 min at room temperature, as recommended by the Monolith His-Tag Labeling Kit RED-tris-NTA protocol (NanoTemper Technologies, MO-L008). PBS was used as the binding buffer for reactions, and 16-step, twofold dilution curves for metformin at the concentrations indicated were created. Labeled protein in binding buffer was then added to diluted metformin or berberine at room temperature. The samples were loaded into standard glass capillaries (NanoTemper Technologies, MO-K022). MST was completed in three independent experiments on a Monolith NT.115 instrument (NanoTemper Technologies) running MO. Affinity Analysis software (v.2.1.23333) with settings of 80% excitation power and 40% MST power at room temperature.

The raw data were obtained from MO. Affinity Analysis software. The fluorescence intensity values were averaged, and the results are expressed as the relative changes from the values in the 0 μM ligand condition. The data were fitted to saturation binding equations using GraphPad Prism 8.0. The dotted lines indicate areas where data could not be fitted.

Histological analysis

Formalin-fixed liver tissue was processed, and 5-μm-thick paraffin sections were stained with hematoxylin and eosin (HE) and Oil Red O (ORO) solution for histological

analysis. The histological examination was performed using the histological scoring system for NAFLD by an experienced pathologist without prior knowledge of the treatments. The NAFLD activity score (NAS) was quantified by summing the scores of steatosis (0-3), lobular inflammation (0-3), and hepatocyte ballooning (0-2). NASH was defined for cases with NASs ≥ 4.

Microarray analyses

Total RNA was extracted and purified using a RNeasy microkit according to the manufacturer's instructions and checked for a RIN number to inspect RNA integration with an Agilent Bioanalyzer 2100 (Agilent Technologies). Total RNA was amplified, labeled and purified using Agilent G3 Mouse GE v2 8×60K according to the manufacturer's instructions at Shanghai Biotechnology Corporation. Data were extracted with Feature Extraction software 10.7 (Agilent Technologies). Raw data were normalized by the quantile algorithm and limma packages in R. The data were analyzed by a bioinformatics analysis service at SHBIO (Shanghai, China). The criteria for differential expression were a $P < 0.05$ and a fold change>±2 relative to the control expression. The DEGs were subjected to GO and KEGG pathway analyses by SHBIO (Shanghai, China) to investigate the potential functions of the DEGs. Pathways with $P < 0.05$ were considered significantly enriched.

References

1 Borra, R. J. *et al.* Nonalcoholic fatty liver disease: rapid evaluation of liver fat content with in-phase and out-of-phase MR imaging. *Radiology* **250**, 130-136, doi:10.1148/radiol.2501071934 (2009).

2 Fishbein, M. H., Gardner, K. G., Potter, C. J., Schmalbrock, P. & Smith, M. A. Introduction of fast MR imaging in the assessment of hepatic steatosis. *Magn Reson Imaging* **15**, 287-293, doi:10.1016/s0730-725x(96)00224-x (1997).

3 Cioffi, C. E. *et al.* Hepatic fat is a stronger correlate of key clinical and molecular abnormalities than visceral and abdominal subcutaneous fat in youth. *BMJ Open Diabetes Res Care* **8**, doi:10.1136/bmjdrc-2019-001126 (2020).

4 Pan, X., Eathiraj, S., Munson, M. & Lambright, D. G. TBC-domain GAPs for Rab GTPases accelerate GTP hydrolysis by a dual-finger mechanism. *Nature* **442**, 303-306, doi:10.1038/nature04847 (2006).

5 Jerabek-Willemsen, M., Wienken, C. J., Braun, D., Baaske, P. & Duhr, S. Molecular interaction studies using microscale thermophoresis. *Assay Drug Dev Technol* **9**, 342-353, doi:10.1089/adt.2011.0380 (2011).

6 Feng, K. *et al.* Hepatic Lipidomics Analysis Reveals the Antiobesity and Cholesterol-Lowering Effects of Tangeretin in High-Fat Diet-Fed Rats. *J Agric Food Chem* **68**, 6142-6153, doi:10.1021/acs.jafc.0c01778 (2020).

7 Qin, M. *et al.* Insights into the prognosis of lipidomic dysregulation for death risk in patients with coronary artery disease. *Clin Transl Med* **10**, e189, doi:10.1002/ctm2.189 (2020).

8 Yuan, X. *et al.* Lipidomics profiling of goose granulosa cell model of stearoyl-CoA desaturase function identifies a pattern of lipid droplets associated with follicle development. *Cell Biosci* **11**, 95, doi:10.1186/s13578-021-00604-6 (2021).

9 Matyash, V., Liebisch, G., Kurzchalia, T. V., Shevchenko, A. & Schwudke, D. Lipid extraction by methyl-tert-butyl ether for high-throughput lipidomics. *J Lipid Res* **49**, 1137-1146, doi:10.1194/jlr.D700041-JLR200 (2008).
