# Supplementary material for: GP73 is a TBC-domain Rab GTPase-activating protein contributing to the pathogenesis of non-alcoholic fatty liver disease without obesity
Source: Nat Commun. 2021 Dec 1;12:7004. doi: 10.1038/s41467-021-27309-1 (PMC8636488; doi:10.1038/s41467-021-27309-1)
Supplement: Supplementary file 3 — Reporting summary. [file 41467_2021_27309_MOESM3_ESM.pdf]

nature research

Corresponding author(s): Yi Fang, Xiaoli Yang, Qi Gao, Feixiang Wu, Hui Zhong, and Congwen Wei

Last updated by author(s): Sep 30, 2021

Reporting Summary

Nature Research wishes to improve the reproducibility of the work that we publish. This form provides structure for consistency and transparency in reporting. For further information on Nature Research policies, see our Editorial Policies and the Editorial Policy Checklist.

Statistics

For all statistical analyses, confirm that the following items are present in the figure legend, table legend, main text, or Methods section.

n/a	Confirmed	
☐	☒	The exact sample size (n) for each experimental group/condition, given as a discrete number and unit of measurement
☐	☒	A statement on whether measurements were taken from distinct samples or whether the same sample was measured repeatedly
☐	☒	The statistical test(s) used AND whether they are one- or two-sided<br>*Only common tests should be described solely by name; describe more complex techniques in the Methods section.*
☒	☐	A description of all covariates tested
☒	☐	A description of any assumptions or corrections, such as tests of normality and adjustment for multiple comparisons
☐	☒	A full description of the statistical parameters including central tendency (e.g. means) or other basic estimates (e.g. regression coefficient) AND variation (e.g. standard deviation) or associated estimates of uncertainty (e.g. confidence intervals)
☐	☒	For null hypothesis testing, the test statistic (e.g. F, t, r) with confidence intervals, effect sizes, degrees of freedom and P value noted<br>*Give P values as exact values whenever suitable.*
☒	☐	For Bayesian analysis, information on the choice of priors and Markov chain Monte Carlo settings
☒	☐	For hierarchical and complex designs, identification of the appropriate level for tests and full reporting of outcomes
☒	☐	Estimates of effect sizes (e.g. Cohen's d, Pearson's r), indicating how they were calculated

Our web collection on statistics for biologists contains articles on many of the points above.

Software and code

Policy information about availability of computer code

Data collection	MO Affinity Analysis (version 2.1.23333), Tecan Spark Control (version 2.1), Tanon Gelcap (version 5.22), QuantStudio Design & Analysis Software (version 1.51), Siemens parallel processing workstation viewing software(version MR B17), Nikon NIS-Elements viewer (version 4.20),
Data analysis	GraphPad Prism (version 8.0), Volocity Demo (Version 6.1.1), Microsoft Office Standard 2010 (version 14.0.4760.1000), Volocity Demo (Version 6.1.1), Nikon NIS-Elements AR (version 4.00.12)

For manuscripts utilizing custom algorithms or software that are central to the research but not yet described in published literature, software must be made available to editors and reviewers. We strongly encourage code deposition in a community repository (e.g. GitHub). See the Nature Research guidelines for submitting code & software for further information.

Data

Policy information about availability of data

All manuscripts must include a data availability statement. This statement should provide the following information, where applicable:

- Accession codes, unique identifiers, or web links for publicly available datasets
- A list of figures that have associated raw data
- A description of any restrictions on data availability

Gene expression microarray data generated for this manuscript have been deposited in the Gene Expression Omnibus database (https://www.ncbi.nlm.nih.gov/geo) under accession number GSE163918. The data that support the findings of this study are available from the corresponding author. Source data are provided with this paper.

Field-specific reporting

Please select the one below that is the best fit for your research. If you are not sure, read the appropriate sections before making your selection.

- [x] Life sciences
- [ ] Behavioural & social sciences
- [ ] Ecological, evolutionary & environmental sciences

For a reference copy of the document with all sections, see nature.com/documents/nr-reporting-summary-flat.pdf

Life sciences study design

All studies must disclose on these points even when the disclosure is negative.

Sample size	In mice experiment, no statistical methods were used to predetermine sample size. We used n=6 in each group based on prior publications using comparable methods. The sample size for human studies we set is n=14 in each group. We then calculated the power by using PASS (version 15.0.5) and the majority of the results was over 0.8. The sample size (n) of each experiment is provided in the corresponding figure captions in the main manuscript and supplementary information files. Sample sizes were chosen to support meaningful conclusions.
Data exclusions	No data were excluded.
Replication	All experimental findings were reproduced a minimum of 2-3 times and all replication attempts were successful. The MST assays were repeated at least three independent times, all attempts at replication were successful. The GAP assays were repeated at least three independent times, all attempts at replication were successful. The ELISA assays were repeated at least two independent times, all attempts at replication were successful. The western blot and immunoprecipitation assays were repeated at least two independent times, all attempts at replication were successful. The qPCR was performed one time in triplicate all attempts at replication were successful. The confocal assays performed at least two independent times, all attempts at replication were successful.
Randomization	No randomization for experimental groups was used in this study. Animal experiments were age-matched and housed in the same cages for the duration. Human study were age-matched and full cohort characteristics are provided in supplementary table 1.
Blinding	The researchers were blinded during the data collection and analysis. Technicians were not blinded to group allocation, as they were not informed of expected treatment results before and during experimentation. Students blinded their experiments through random numbering of treatment groups and the code was unblinded after analysis. As clinical characteristics for all individuals enrolled in this study are necessary to undergo MRI, no blinding was performed. Full cohort characteristics are provided in supplementary table 1.

Reporting for specific materials, systems and methods

We require information from authors about some types of materials, experimental systems and methods used in many studies. Here, indicate whether each material, system or method listed is relevant to your study. If you are not sure if a list item applies to your research, read the appropriate section before selecting a response.

Materials & experimental systems

n/a	Involved in the study	
[ ]	[x]	Antibodies
[ ]	[x]	Eukaryotic cell lines
[x]	[ ]	Palaeontology and archaeology
[ ]	[x]	Animals and other organisms
[ ]	[x]	Human research participants
[x]	[ ]	Clinical data
[x]	[ ]	Dual use research of concern

Methods

n/a	Involved in the study	
[x]	[ ]	ChIP-seq
[x]	[ ]	Flow cytometry
[x]	[ ]	MRI-based neuroimaging

Antibodies

Antibodies used	Anti-α-Tubulin for western blot (Supplier: Proteintech, Cat: 11224-1-AP, Lot: 00084996)<br>Anti-GP73 for western blot (Supplier: Abcam, Cat: ab92612, clone: OTI6C9, Lot: GR3250053-6)<br>Anti-ApoB for western blot (Supplier: Proteintech Group, Cat: 20578-1-AP, Lot: 00025612)<br>Anti-mouse-Flag for western blot (Supplier: Sigma-Aldrich, Cat: F1804, Lot: SLBF6631)<br>Anti-mouse-Myc for western blot (Supplier: ABclonal, Cat: AE010, Lot: 4000049011)<br>Anti-GP73 for immunofluorescence (Supplier: BOSTER, Cat: A02975-2, Lot: BOS7542BP4109)<br>Anti-Albumin for immunofluorescence (Supplier: Abclonal, Cat: A0353, Lot: 0202160201)
Validation	All antibodies sourced from commercial corporation are well-validated by the manufacturer and are widely used in the scientific community for Western blotting.<br>Anti-α-Tubulin for western blot (Supplier: Proteintech, Cat: 11224-1-AP)<br>https://www.ptglab.com/products/TUBA1B-Antibody-11224-1-AP.htm

Anti-GP73 for western blot (Supplier: Abcam, Cat: ab92612, clone: OTI6C9)
https://www.abcam.com/golph2-antibody-ab92612.html

Anti-ApoB for western blot (Supplier: Proteintech Group, Cat: 20578-1-AP)
https://www.ptglab.com/products/APOB-Antibody-20578-1-AP.htm

Anti-mouse-Flag for western blot (Supplier: Sigma-Aldrich, Cat: F1804)
https://www.sigmaaldrich.com/catalog/product/sigma/f1804

Anti-mouse-Myc for western blot (Supplier: ABclonal, Cat: AE010)
https://abclonal.com.cn/catalog/AE010

Anti-GP73 for immunofluorescence (Supplier: BOSTER, Cat: A02975-2)
http://www.boster.com.cn/product/anti-golm1-antibody_a02975-2.html

Anti-Albumin for immunofluorescence (Supplier: Abclonal, Cat: A0353)
https://abclonal.com.cn/catalog/A0353

Eukaryotic cell lines

Policy information about cell lines

Cell line source(s)	293T (CRL-3216) cell lines were from the American Type Culture Collection (ATCC, Rockville, MD, USA). Huh-7 (0403) was from Japanese Collection of Research Bioresources.
Authentication	No authentication was performed on cell lines.
Mycoplasma contamination	Cell lines tested mycoplasma contamination periodically and cell lines tested negative.
Commonly misidentified lines (See ICLAC register)	No commonly misidentified lines were used.

Animals and other organisms

Policy information about studies involving animals; ARRIVE guidelines recommended for reporting animal research

Laboratory animals	All mice used in this study were stated in method and figure legends. Male C57BL/6N mice (6–8 weeks of age) were purchased from SPF Biotechnology (Beijing, China).<br>All mice were housed in colony cages in a pathogen-free environment with the temperature maintained at 21–23 °C and relative humidity at 50–60%, and were under a 12 hr light/12 hr dark cycle. All mice were fed ad libitum with standard chow diet (Teklad 2919, 9% fat) prior to study. Six-to-eight weeks old male mice were used in our experiments.
Wild animals	The study did not involve wild animals.
Field-collected samples	The study did not involve field-collected samples.
Ethics oversight	All animal experiments were conducted at the AMMS Animal Center (Beijing, China) and were approved by the Institutional Animal Care and Use Committee.

Note that full information on the approval of the study protocol must also be provided in the manuscript.

Human research participants

Policy information about studies involving human research participants

Population characteristics	Fourteen patients with NAFLD without obesity and 14 non-obese healthy comparison subjects with normal liver on MRI were included in the study. NAFLD with <25 kg/m/m BMI was defined as NAFLD without obesity. All the patients had no history of any hepatotoxic drugs, hormone replacement therapy, or herbal products and consumed no more than 20 g/day alcohol. The healthy control group had no illness, no usage of alcohol, drugs, or herbal substances, no history of previous liver diseases, and was negative for viral hepatitis serology tests and had normal liver. Clinical characteristics of the human research participants was provided in Supplementary Table 1.
Recruitment	All human blood samples and clinical information were obtained from the Third Medical Center of the Chinese PLA General Hospital. Informed consent was obtained from participants included in this study. Fourteen non-obese healthy subjects and 14 NAFLD without obesity subjects diagnosed with MRI were included in the study. These participants were recruited randomly from the physical examination population, so there was no potential self-selection bias or other biases that may be present and impact results.
Ethics oversight	The Committee for Ethics in Human Studies from the Third Medical Center of the Chinese PLA General Hospital approved this study (KY2021-009).

Note that full information on the approval of the study protocol must also be provided in the manuscript.
